# A comprehensive study of risk factors for post-operative pneumonia following resection of meningioma

**DOI:** 10.1186/s12885-019-5271-7

**Published:** 2019-01-23

**Authors:** M. R. Zuo, R. F. Liang, M. Li, Y. F. Xiang, S. X. Zhang, Y. Yang, X. Wang, Q. Mao, Y. H. Liu

**Affiliations:** Department of Neurosurgery, West China Hospital, Sichuan University, Cheng Du, 610000 China

**Keywords:** Post-operative pneumonia, Hematological indicators, Risk factors, Meningioma

## Abstract

**Background:**

Post-operative pneumonia (Pop) following meningioma surgery is the dominant systemic complication which could cause serious threats to patients. It is unclear whether hematological biochemical markers are independently associated with the Pop. This study attempted to perform a more comprehensive study of taking both clinical factors and hematological biomarkers into account to promote the management of patients after meningioma surgery.

**Methods:**

We collected clinical and hematological parameters of 1156 patients undergoing meningioma resection from January 2009 to January 2013. According to whether the symptoms of pneumonia had manifested,patients were divided into the Pop group and the Non-Pop group. We analyzed the distinctions of clinical factors between the two groups. We successively performed univariate and multivariate regression analysis to identify risk factors independently associated with the Pop.

**Results:**

4.4% patients infected with the Pop (51 of 1156). The median age at diagnosis of the Pop patients was significantly older than the Non-Pop group (*p* = 0.002). There were strike distinctions of post-operative hospital stays between two groups, with 21 days and 7 days each (*p* < 0.001). On multivariate analysis, tumor relapse (*p* < 0.001), skull base lesions (*p* = 0.001), intra-operative blood transfusion (*p* = 0.018) and cardiovascular diseases (*p* = 0.001) were linked with increased risk of the Pop following meningioma resection. For hematological biochemical markers, it was the factor of Red blood cell distribution width-standard deviation (RDW-SD) (OR 5.267, 95%CI 1.316, 21.078; *p* = 0.019) and Neutrophils lymphocytes ratio (NLR) (OR 2.081, 95%CI 1.063, 4.067; *p* = 0.033) that could appreciably predict the Pop.

**Conclusions:**

Apart from tumor recurrence, localizations, intra-operative blood transfusion and cardiovascular diseases are independent risk factors for the Pop. We initially found hematological RDW-SD and NLR are also important predictors.

## Background

Meningiomas are considered as the most common brain tumor with a reported morbidity of 35% according to Central Brain Tumor Registry of the United States (CBTRUS) in 2006–2010. Meanwhile, about 98% of meningiomas were identified to be with non-malignant histopathological features [1]. Although it can be cured by total resection, approximately 10–30% of patients obtaining total lesion resection and 60% of patients with subtotal tumor removal would suffer a relapse within 10 years [2, 3]. It was also noteworthy that anaplastic meningiomas were associated with poorer overall survival for its higher possibility of local invasion and recurrence [4–6]. In addition to risks of tumor itself, acquired post-operative complications were other factors causing a heavy physical and mental burden to patients, and even lead to mortality [7]. Apart from those well-known post-operative surgical and neurological complications [3, 8, 9], several serious medical complications occurring in other systems following meningioma resection had rarely been studied systematically.

Post-operative pneumonia (Pop) as one of the medical complications usually occurs with an increased morbidity after surgical treatment [10, 11]. A large cohort study exploring the medical complications following meningioma removal in a single institution showed that the Pop was the leading medical complication with a proportion of 1.3%, [7] which was much less than the reported rates of below 15% [12–14]. The Pop would pose serious threat in the rehabilitation of patients, which may include delayed discharge from hospital, the necessity of intensive care and ventilation or tracheotomy, increased cost of treatment and higher infection related mortality. [15, 16]

It is the prevention of the Pop that should become the top priority in post-operative care, so thorough pinpointing potential risk factors for the Pop should be warranted [7]. Several studies had already devoted to identify the risk factors independently associated with the Pop, which included older age, partial tumor resection, procedure duration and so forth [14, 17, 18]. Furthermore, pre-operative hematological biomarkers had also been verified to be associated with the Pop, such as lower serum albumin, C-reaction protein (CRP) and red blood cell distribution width (RDW) and so on [19–21].

However, the effect of the pretreatment hematological biomarkers on the Pop following meningioma surgery has rarely been detected. This study attempted to investigate if pre-operative hematological biochemical markers were associated with the Pop. We had performed a comprehensive study of taking both clinical factors and hematological biomarkers into account. Identifying thoroughly featured risk factors for the Pop of meningiomas would direct prophylactic measures and ameliorate the results of the Pop.

## Methods

### Patient enrollment

**Fig. 1:**
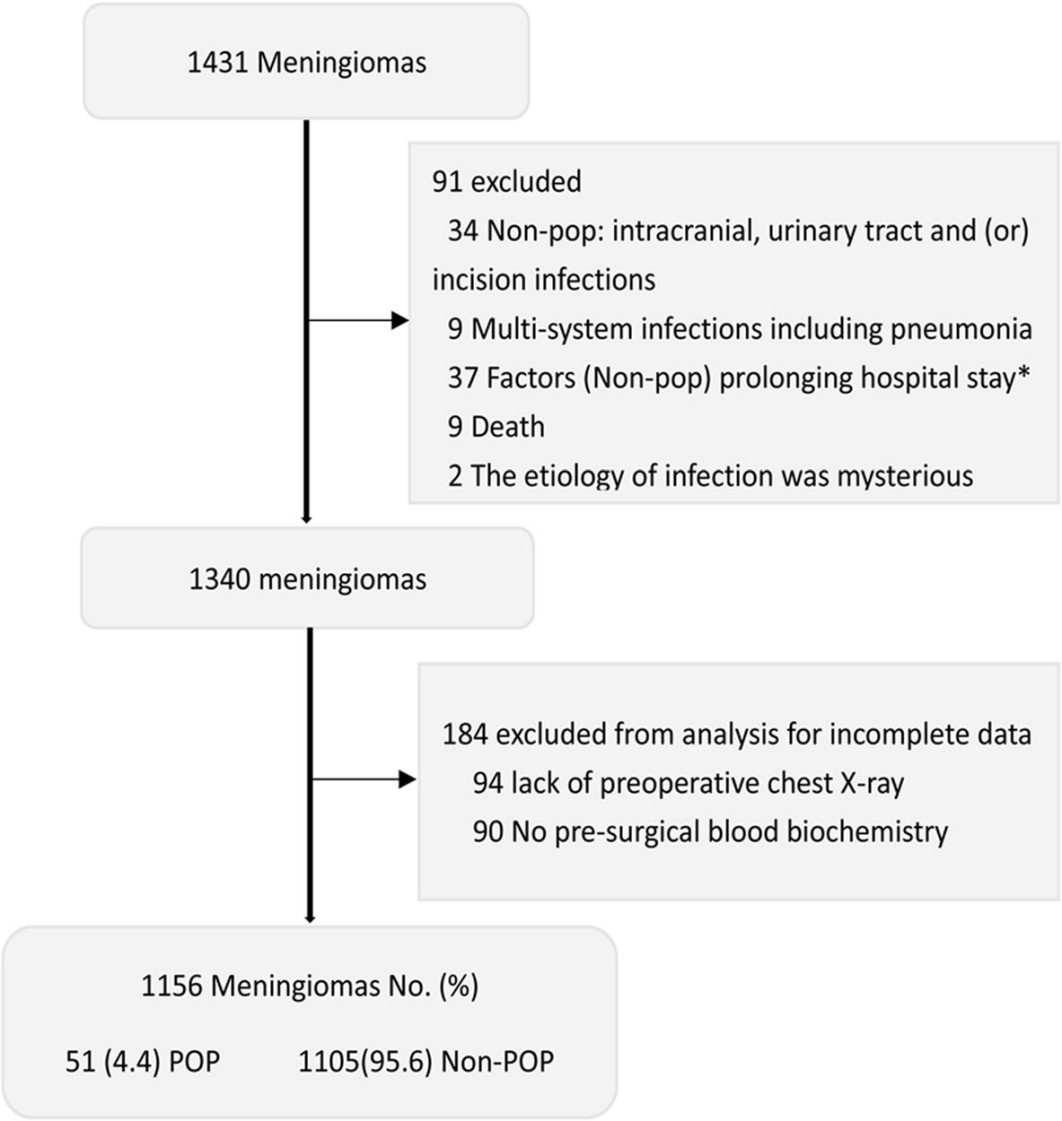
Flow Diagram Demonstrating the Criteria of Exclusion for Patients Undergoing Meningioma Resection. *: Post-operative Hydrocephalus, Subcutaneous Effusion, Hematoma Evacuation, Cerebral Spinal Fluid (CSF) Leakage, Epilepsy, Hypothalamic Syndrome, Tracheotomy, Gastrointestinal Bleeding, Mental Disorder, Arrhythmia, Multi-Organ Tumors Resection, Drug Side Effects, Rehabilitation

We retrospectively reviewed 1431 patients who underwent meningioma resection at West China Hospital from January 1, 2009 to January 30, 2013. These tumors were histologically diagnosed with meningiomas (WHO grade I,II,III) by the pathologic department in our hospital. In the present study, there were two exclusion criteria (Fig. 1): First, we excluded 91 patients who had longer or abnormal hospital stay owing to any reasons had nothing to do with the Pop. Second, we precluded another 184 patients from analysis for incomplete clinical data. In our study, 1156 patients’ information were eligible for analysis, of which 51 patients infected with the Pop (4.4%, 51 in 1156) and the remaining 1105 patients were belong to the group of the Non-postoperative pneumonia (Non-Pop) (Fig. 1). This study was approved by our institutional ethics committee.

### Definition of the post-operative pneumonia

Patients were diagnosed with the Pop suggesting an infection of single or both lungs according to the following post-operative radiological and clinical findings within 30 days.

The radiological standards should include a definitive chest X-ray or CT examination representing at least one of the following: new or progressive and consistent infiltration, opacity or consolidation, and cavitation. The clinical symptoms needed to meet one of the following conditions: First, existence new or progressive and continuous coughing and expectoration. Second, fever (> 38 °C) with no other recognized cause and an abnormal of white blood cell counts: leukopenia (< 4000/mm^3^) or leukocytosis (≥12,000/mm^3^). Third, positive respiratory cultures from sputum or blood [22–24].

### Data collection and study variables

The clinical materials of all patients were obtained from the Hospital Information System (HIS) and were carefully recorded. We firstly analyzed variables as follow: age at diagnosis, gender, relapse, tumor localizations (Skull base and Non-skull base) [25], grades, preoperative steroids administration and chest X-ray, smoking history, type II diabetes mellitus (DM), cardiovascular disease (CVD: coronary heart disease, hypertension, arrhythmia and prior myocardial infarction), blood transfusion during surgery, maximum lesion diameter by one dimension (cm), post-operative hospital stays (days) and pretreatment hematological biochemical markers performed at the time of admission. The specific information of those variables are listed in Tables 1 and 2, respectively. Patients were considered as smokers if they remained smoking frequently on admission day [18]. Preoperative chest X-ray or CT scans demonstrated one of signs of lungs as follow: strikingly increased pulmonary markings, nodular opacities, pulmonary bullous and /or emphysema and signs of chronic bronchitis were defined as abnormality [26]. In regression analysis, age was divided into dichotomous variables according to 65 years. For patients with preoperative steroids administration, the laboratory examinations were performed prior to their medication.

**Table 1:** Demographic Data of 1156 Patients Undergoing Meningioma Resection

Variables	Pneumonia		No pneumonia		
	(*n* = 51)		(*n* = 1105)		
	Median(IQR)	Range	Range	Median(IQR)	*P* Value
Age at diagnosis, years	58 (49~66)	24–81	18–85	51 (43~61)	0.002^a^
Gender No. (%)					
Female	34 (67)			782 (71)	0.530^b^
Male	17 (33)			323 (29)	
Recurrent					
Yes	8 (16)			41 (4)	< 0.001^b^
No	43 (84)			1064 (96)	
Tumor localizations					
Skull base	27 (53)			394 (36)	0.003^b^
Non-skull base ^c^	24 (47)			711 (64)	
Grade					
I	41 (80)			906 (82)	0.772^b^
II/III	10 (20)			199 (18)	
Preoperative Steroids					
Yes	8 (16)			96 (9)	0.125^b^
No	43 (84)			1009 (91)	
Preoperative Chest X-ray					
Normal	28 (55)			767 (69)	0.029^b^
Abnormal	23 (45)			338 (31)	
Smoking					
Yes	10 (20)			132 (12)	0.103^b^
No	41 (80)			973 (88)	
DM					
Yes	4 (8)			36 (3)	0.095^b^
No	47 (92)			1069 (97)	
CVD					
Yes	13 (25)			111 (10)	< 0.001^b^
No	38 (75)			994 (90)	
Blood transfusion					
Yes	10 (20)			80 (7)	0.004^b^
No	41 (80)			1025 (93)	
Size (cm)					
< 3 cm	3 (6)			167 (15)	0.006^b^
3 cm–5 cm	14 (27)			439 (40)	
≥ 5 cm	34 (67)			483 (44)	
Postoperative hospital stay, days	21 (17~29)	7–229	2~20	7 (6~9)	< 0.001^a^

Non-normally distributed numerical variables are represented as median and Interquartile range (IQR)

^a^Wilcoxon–Mann–Whitney test. ^b^Fisher’s exact test

^c^Convexity, falx/parasagittal, cerebellar convexity and intra-ventricular

Skull base: Cerebellopontine angle, foramen magnum, middle fossa, olfactory groove/planum shenoidale, orbital/ anterior clinoid, parasellar/cavernous sinus, petroclival, sphenoid wing, tentorium and tuberculum sellae

**Table 2:** Preoperative Data of Hematological Biochemical Markers of 1156 Patients Undergoing Meningioma Resection

Variables		Pneumonia (*n* = 51)		Non-pneumonia (*n* = 1105)	
		Value		Value	
HGB	(< 110/110~150/> 150 g/L)	3/42/6	130(IQR 119~140)	61/901/143	133(IQR 123~143)
HCT	(< 0.36/0.36~0.47/> 0.47 L/L)	6/43/2	0.41(IQR 0.37~0.43)	86/960/59	0.41(IQR 0.38~0.44)
MCV	(< 80/≥80 fL)	3/48	90.7(IQR 88~94.7)	69/1036	91.3(IQR 88.3~94.2)
MCH	(< 27/27~32/> 32 pg)	6/41/4	29.6(IQR 28.3~30.6)	106/906/93	29.9(IQR 28.8~30.9)
MCHC	(< 310/≥310 g/L)	9/42	324(IQR 315~332)	96/1009	326(IQR 319~333)
RDW-SD	(≤54/> 54 fL)	46/5	46.2(IQR 43.3~49.4)	1095/10	45.1(IQR 42.9~47.25)
RDW-CV	(≤14.5/> 14.5%)	37/14	14(IQR 13.2~14.9)	929/176	13.6(IQR 13.1~14.2)
Platelet Counts	(< 100/≥100 × 10^9^/L)	6/45	174(IQR 137~214)	60/1045	174(IQR 139~217.5)
WBC	(< 4/4~10/ > 10 × 10^9^/L)	4/38/9	6.28(IQR 5.44~8)	76/953/76	6(IQR 5.01~7.295)
Neutrophils	(< 2.0/2.0~7.5/> 7.5 × 10^9^/L)	1/42/8	4.17(IQR 3.27~6.11)	35/991/79	3.75(IQR 2.97~4.995)
Lymphocytes	(< 3.5/≥3.5 × 10^9^/L)	2/49	1.59(IQR 1.12~1.79)	1095/10	1.62(IQR 1.25~2)
Monocytes	(< 0.12/≥0.12 × 10^9^/L)	7/44	0.3(IQR 0.2~0.4)	59/1046	0.29(IQR 0.22~0.37)
Albumin	(< 35/≥35 g/L)	4/47	41.9(IQR 39.1~43.8)	28/1077	42.9(IQR 40.5~45.2)
Globulin	(≤34/> 34 g/L)	7/44	27.5(IQR 25.1~31.7)	1039/66	27.1(IQR 24.4~29.8)
TG	(≤1.83/> 1.83 mmol/L)	11/40	1.18(IQR 0.84~1.78)	844/261	1.24(IQR 0.905~1.78)
Cholesterol	(≤5.7/> 5.7 mmol/L)	43/8	4.67(IQR 1.08~5.47)	942/163	4.66(IQR 4.07~5.29)
HDL	(≤0.9/> 0.9 mmol/L)	2/49	1.33(IQR 1.18~1.55)	64/1041	1.4(IQR 1.17~1.67)
A/G	(< 1.2/≥1.2)	8/43	1.54(IQR 1.3~1.7)	56/1049	1.58(IQR 1.41~1.77)
NLR	(< 2.5/2.5~5/> 5)	19/22/10	2.73(IQR 2.1~4.72)	633/343/129	2.27(IQR 1.755~3.24)

Non-normally distributed numerical variables are represented as median and Interquartile range (IQR)

*HGB* Hemoglobin, *HCT* Hematocrit, *MCV* Mean corpuscular volume, *MCHC* Mean corpuscular hemoglobin concentration, *RDW-SD* Red blood cell distribution width-standard deviation, *RDW-CV* Red blood cell distribution width-coefficient of variation, *WBC* White blood cell, *TG* Triglyceride, *HDL* High density lipoprotein, *A/G* Albumin/Globulin, *NLR* Neutrophils lymphocytes ratio

### Statistical analysis

Patients were separately classified into the Pop group and the Non-Pop group. The Kolmogorov-Smirnov test was performed to define if variables were normally distributed. Non-normally distributed variables were represented as median and Interquartile range (IQR). Distinctions of continuous variables between groups were compared by Mann-Whitney U test and a chi-square test or Fisher exact test was performed for categorical variables. Risk factors that might independently affect the occurrence of the Pop were analyzed by using univariate logistic regression and a *p* < 0.05 was required for further multivariate analysis to test the association of combined predictors with the Pop outcome. Statistical analysis was conducted by using SPSS version 20 for Windows (SPSS Inc). *P* < 0.05 was considered as statistically significant.

## Results

### Demographic data of 1156 patients with meningioma resection

In this cohort, the frequency of the Pop following meningioma surgery was 4.4% (51 in 1156 patients). The median age at diagnosis of the Pop was 58-years old [Interquartile range (IQR) 49, 66]. On the contrary, the median age of the Non-Pop patients, 51-years old (43, 61), was significantly younger than the previous group (*p* = 0.002). The Pop patients following meningioma resection seemed likely to be featured on those factors, such as tumor recurrence, skull base lesions, cardio-vascular diseases, intra-operative blood transfusion as compared to the Non-Pop patients. There were appreciable distinctions of post-operative hospital stays between the two groups, with 21 days (IQR 17, 29) and 7 days (IQR 6, 9) each (*p* < 0.001). The specific clinical information and distinctions between the two groups are listed in Table 1.

For all 51 patients with the Pop following meningioma resection, of which 16 cases were confirmed by systematically evaluating the clinical symptoms, laboratory tests and radiology, although no microbial culture. With regard to positive respiratory cultures, 11 patients had sputum cultures with a growth of *Klebsiella pneumoniae* and 4 patients with positive cultures of Pseudomonas aeruginosa. Eight of those patients had Bowman’s / Acinetobacter selenium complex, 6 with *Escherichia coli*, 4 with *Candida albicans*, 4 with *Enterobacter aerogenes*, 4 with *Staphylococcus aureus*, 2 with Proteus mirabilis, 2 with Hemolytic *Staphylococcus aureus*, another 1 with Serratia marcescens positivity, respectively. There were 11 patients with more than two kinds of bacterial growth.

### Pre-operative data of hematological biochemical markers

We included 19 hematological biochemical markers which all derived from the department of laboratory medicine of our hospital. All markers were stratified into dichotomous or trichotomous variables on the basis of the standard ranges of our clinic center and the actual distribution of each factor. To analyze HGB, HCT, MCH, WBC, Neutrophils and NLR (neutrophil lymphocyte ratio) by using trichotomy. For the rest of markers, a traditional dichotomous method was taken. The specific data are listed in Table 2.

### Univariate and multivariate logistic regression analysis for risk factors for the pop

Univariate logistic regression analysis was conducted to select factors that may be linked with the Pop following meningioma resection. The threshold of each factor was consistent with the classification as mentioned in Table 2. In this study, aged patients (Odds ratio 2.509, 95% Confidence Interval 1.105, 3.837), relapse (OR 4.828, 95% CI 2.134, 10.925), skull base localizations (OR 2.325,95% CI 1.308, 4.131), cardiovascular diseases (OR 3.064, 95% CI 1.584, 5.925), intra-operative blood transfusion (OR 3.125, 95% CI 1.509, 6.470), lower MCHC (OR 0.454, 95% CI 0.215, 0.961), higher RDW-SD (OR 11.902, 95% CI 3.91, 36.235), higher RDW-CV (OR 1.997, 95% CI 1.158, 3.772), lower monocytes (OR 0.355, 95% CI 0.153, 0.821), lower albumin (OR 0.305, 95% CI 1.103, 0.906, higher Globulin (OR 2.504, 95% CI 1.086, 5.775)), lower A/G (OR 0.287, 95% CI 0.129, 0.639) and higher NLR (OR 2.137, 95% CI 1.141, 4.003) were identified as risk factors associated with the Pop (Table 3).

**Table 3:** Univariate and Multivariate Cox Regression Analysis of Risk Factors for Pop after Meningioma Resection

Variables	Univariate Cox Regression		Multivariate Cox Regression	
	Odds Ratio(95%CI)	*P* Value	Odds Ratio(95%CI)	*P* Value
Age (< 65-years old)	2.509 (1.105~3.837)	0.023		
Gender (Female)	1.211 (0.667~2.198)	0.53		
Recurrent (No)	4.828 (2.134~10.925)	< 0.001	7.013 (2.772~17.74)	< 0.001
Localizations (Non-skull base)	2.325 (1.308~4.131)	0.004	2.784 (1.482~5.229)	0.001
Grades (I)				
Grade II	0.988 (0.455~2.142)	0.975		
Grade III	2.210 (0.500~9.774)	0.296		
Preoperative Steroids (No)	1.955 (0.894~4.279)	0.093		
Chest-X Ray (Normal)	1.900 (0.901~4.006)	0.092		
Smoking (No)	1.798 (0.88~3.674)	0.108		
DM (No)	2.257 (0.864~7.394)	0.091		
CVD (No)	3.064 (1.584~5.925)	0.001	3.692 (1.734~7.862)	0.001
Blood Transfusion (No)	3.125 (1.509~6.470)	0.002	2.659 (1.180~5.992)	0.018
Size (< 5 cm)	2.878 (0.886~9.347)	0.079		
HGB (< 110 g/L)				
110~150 g/L	0.948 (0.286~3.146)	0.930		
> 150 g/L	0.853 (0.207~3.522)	0.826		
HCT (< 0.36 L/L)				
0.36~0.47 L/L	0.642 (0.266~1.551)	0.325		
> 0.47 L/L	0.486 (0.095~2.490)	0.387		
MCV(< 80 fl)	1.066 (0.324~3.508)	0.917		
MCH (< 27 pg)				
27~32 pg	0.792 (0.328~1.910)	0.603		
> 32 pg	0.745 (0.204~2.720)	0.656		
MCHC (< 310 g/L)	0.454 (0.215~0.961)	0.039		
RDW-SD(≤54 fl)	11.902 (3.91~36.235)	< 0.001	5.267 (1.316~21.078)	0.019
RDW-CV(≤14.5%)	1.997 (1.158~3.772)	0.033		
Platelet Count(< 100 × 10^9^/L)	0.431 (0.177~1.049)	0.064		
WBC (< 4 × 10^9^/L)				
4~10 × 10^9^/L	0.758 (0.263~2.179)	0.607		
> 10 × 10^9^/L	2.250 (0.664~7.621)	0.193		
Neutrophils (< 2 × 10^9^/L)				
2~7.5 × 10^9^/L	1.483 (0.198~11.088)	0.701		
> 7.5 × 10^9^/L	3.544 (0.427~29.428)	0.241		
Lymphocytes (< 3.5 × 10^9^/L)	4.469 (0.953~20.951)	0.058		
Monocytes (< 0.12 × 10^9^/L)	0.355 (0.153~0.821)	0.015		
Albumin (< 35 g/L)	0.305 (1.103~0.906)	0.033		
Globulin (≤34 g/L)	2.504 (1.086~5.775)	0.031		
TG (≤1.83 mmol/L)	0.966 (0.498~1.871)	0.918		
Cholesterol (≤5.7/mmol/L)	1.083 (0.500~2.346)	0.840		
HDL (≤0.9 mmol/L)	1.333 (0.316~5.618)	0.696		
A/G (< 1.2)	0.287 (0.129~0.639)	0.002		
NLR (< 2.5)				
2.5~5	2.137 (1.141~4.003)	0.018	2.081 (1.063~4.067)	0.033
> 5	2.583 (1.174~5.684)	0.018		

To comprehensively analyze the most valuable predictors for patients suffering the Pop after surgery, a multivariate regression analysis was performed. For all parameters above, a *p*-value < 0.05 on univariate analysis was eligible for the following multivariate analysis. Regarding clinical factors, tumor recurrence (OR 7.013, 95%CI 2.772, 17.74; *p* < 0.001), skull base tumors (OR 2.784, 95%CI 1.482, 5.229; *p* = 0.001), cardiovascular diseases history (OR 3.692, 95%CI 1.734, 7.862; *p* = 0.001) and intra-operative blood transfusion (OR 2.659, 95%CI 1.180, 5.992; *p* = 0.018) were discovered to be significantly associated with the Pop. While concerning about the preoperative hematological biochemical markers, it was the factor of RDW-SD (OR 5.267, 95%CI 1.316, 21.087; *p* = 0.019) and NLR (OR 2.081, 95%CI 1.063, 4.067; *p* = 0.033) that could significantly predict the Pop for meningiomas after surgery. The details are listed in Table 3.

## Discussion

Post-operative pneumonia is highly likely to have a bad influence on patients’ rehabilitation, such as prolonged hospitalization and higher infection related mortality [15, 16]. It is imperative that factors associated with the Pop for meningiomas should be deeply understood. Based on this study, 4.4% patients infected with the Pop (51 of 1156), which appears to be in consistency with previous reported incidences of less than 15% [7, 14, 17, 18, 21, 26, 27]. Several studies reported that patients with the Pop would inevitably suffer significantly prolonged hospital stays, which means increased health care costs [18, 19, 27–29]. Then we conducted a comparison of post-operative hospitalizations between the two groups. We found there were considerable distinctions of post-operative hospital stays, with 21 days and 7 days each. This result was identical to the previous findings. [19] For this sake, it is essential that finding more precise methods to predict and prevent the occurrence of the Pop during peri-operative period. A pilot study suggested that peri-operative oral care should be emphasized to reduce the possibility of the Pop for patients following esophageal cancer treatment [30].

According to the latest risk stratification for meningiomas, it was suggest that the tumor size excess than 5 cm and tumor located in anterior skull base were adversely impacted on the progression free survival [25]. In this study, we adopted the suggestions to detect if those parameters could be utilized to predict the Pop. We identified two factors, relapse (*p* < 0.001) and skull base localizations (*p* = 0.001) were intensely associated with the occurrence of the Pop. It seemed that we initially found the association between the meningioma relapse and the Pop. While another large cohort research reported negative results by only analyzing the effect of previous craniotomy on post-operative complications [7]. It is well known that refractory meningiomas have dismal prognosis so that the therapeutic strategy should be cautious and more prospective studies relating to the relationship between relapsing meningiomas and the Pop should be warranted [31]. Unlike the effect of the larger tumor size on predicting the relapse of meningioma, it had nothing to do with the Pop in this study [18, 25].

We also highly recommended that meningiomas located in the skull base was a significant factor predicating the Pop which was out of accord with the previous study [7]. Due to its complicated anatomy, the skull base meningiomas are prone to relapse after standard treatment, therefore the specific mechanisms need to be elucidated [30]. A study discovered that partial resection of tumors adjacent to brainstem was an independent risk factor of the Pop, which may lead to the rational hypothesis that the minor possibility of total removal of meningiomas infiltrated with critical tissues was one of the reason why those two factors above were associated with the Pop [14].

Regarding the comorbidity of patients with meningiomas, it showed that cardiovascular diseases were linked with increased risk of the Pop (*p* = 0.001), as compared to the meaningless factors such as smoking and preoperative DM in multivariate regression analysis. Several studies also didn’t report the relationship between smoking, DM and the Pop following different kinds of surgical procedures [17, 18, 30]. Although there were contradictions about the relation between smoking and the Pop, we encourage the smoking cessation in order to decrease a wide variety of post-operative complications [14, 32]. Previous study demonstrated that meningioma patients with hypertension or being on cardiac medications were inclined to develop serious medical complications on univariate analysis [7]. According to the present study, we recommended that the condition of cardiovascular system of patients should be carefully evaluated prior to meningioma resection. We also found significant correlation between the intra-operative blood transfusion and the Pop, which was in line with the result in other systemic disease [17]. We suggest that surgeons keep this factor in mind during operations in order to decrease the occurrence of Pop.

Pre-operative laboratory biomarkers have drawn great attention for its prognostic significance in a variety of cancers including meningiomas [33–36]. Apart from its role in predicting survival, it was also demonstrated that preoperative RDW played an important role in predicating the occurrence of the Pop following hip fracture surgery, for instance [21]. Pre-operative serum albumin had been proven to be a significant indicator for wound infection as well [20]. In addition, NLR as an easily accessible parameter, has been considered as a convincible factor for predicting prognosis for several tumors [34, 36]. As far as we know, the significance of hematological biomarkers for predicting the Pop of meningiomas has rarely been reported till now. Thus for these markers, it was the RDW-SD and NLR that could appreciably predict the Pop of meningiomas in the present study (*p* = 0.019 and *p* = 0.033 respectively). It had been elucidated that RDW played an important role in foreseeing age-related illnesses [37, 38]. Elevated RDW values could reflect chronic systemic inflammation and poor nutritional status. We can make a speculation of the elevated RDW-SD could be a potential marker to predict the post-operative medical complications associated with age-related diseases through affecting or altering the overall inner environment and the physiological state. Therefore, the specific mechanism of the effect of RDW on overall health should be investigated deeply and afterward experimental schemes should consider the influence of RDW in order to predict the Pop more accurately. We evaluated the usefulness of NLR in this study for the first time. The outcome suggested that NLR was a significant risk factor for the Pop after meningioma surgery. Combining those two factors above, it reminded us all that careful consideration of pretreatment hematological status and systemic inflammatory 12response played an important role in management of peri-operative meningioma patients.

## Conclusion

In summary, more accurate and comprehensive predictors for the Pop of meningiomas should be sufficiently understood so as to direct management of patients during peri-operation period. Our findings suggested that predictors, such as tumor recurrence, skull base lesions, cardiovascular diseases and intra-operative blood transfusion, are independent risk factors of the Pop for meningioma surgery. Most importantly, we found preoperative hematological RDW-SD and the parameter of NLR, both of them are easily accessible markers from laboratory test, are new predictors for the Pop for meningiomas.

## Abbreviations

A/G: Albumin/Globulin
CBTRUS: Central Brain Tumor Registry of the United States
CI: Confidence Interval
CRP: C-reaction protein
CVD: Cardiovascular disease
DM: Diabetes mellitus
HCT: Hematocrit
HDL: High density lipoprotein
HGB: Hemoglobin
HIS: Hospital Information System
IQR: Interquartile range
MCHC: Mean corpuscular hemoglobin concentration
MCV: Mean corpuscular volume
NLR: Neutrophils lymphocytes ratio
OR: Odds ratio
POP: Post-operative pneumonia
RDW: Red blood cell distribution width
RDW-CV: Red blood cell distribution width-coefficient of variation
RDW-SD: Red blood cell distribution width-standard deviation
TG: Triglyceride
WBC: White blood cell
